# Ethyl 4-butyl­amino-3-nitro­benzoate

**DOI:** 10.1107/S1600536809029754

**Published:** 2009-08-08

**Authors:** Shivanagere Nagojappa Narendra Babu, Aisyah Saad Abdul Rahim, Shafida Abd Hamid, Kasthuri Balasubramani, Hoong-Kun Fun

**Affiliations:** a School of Pharmaceutical Sciences, Universiti Sains Malaysia, 11800 USM, Penang, Malaysia; b Kulliyyah of Science, International Islamic University Malaysia (IIUM), Jalan Istana, Bandar Indera Mahkota, 25200 Kuantan, Pahang, Malaysia; c X-ray Crystallography Unit, School of Physics, Universiti Sains Malaysia, 11800 USM, Penang, Malaysia

## Abstract

In the crystal structure of the title compound, C_13_H_18_N_2_O_4_, the asymmetric unit consists of three crystallographically independent ethyl 4-butyl­amino-3-nitro­benzoate mol­ecules. There is an intra­molecular N—H⋯O hydrogen bond in each mol­ecule, which generates an *S*(6) ring motif. The structure is stabilized by inter­molecular N—H⋯O and C—H⋯O hydrogen bonds.

## Related literature

For nitro­benzoic acid, see: Brouillette *et al.* (1999 ▶); Williams *et al.* (1995 ▶); For benzimdazole derivatives, see Ozden *et al.* (2005 ▶); Beaulieu *et al.* (2004 ▶); Kilburn *et al.* (2000 ▶).
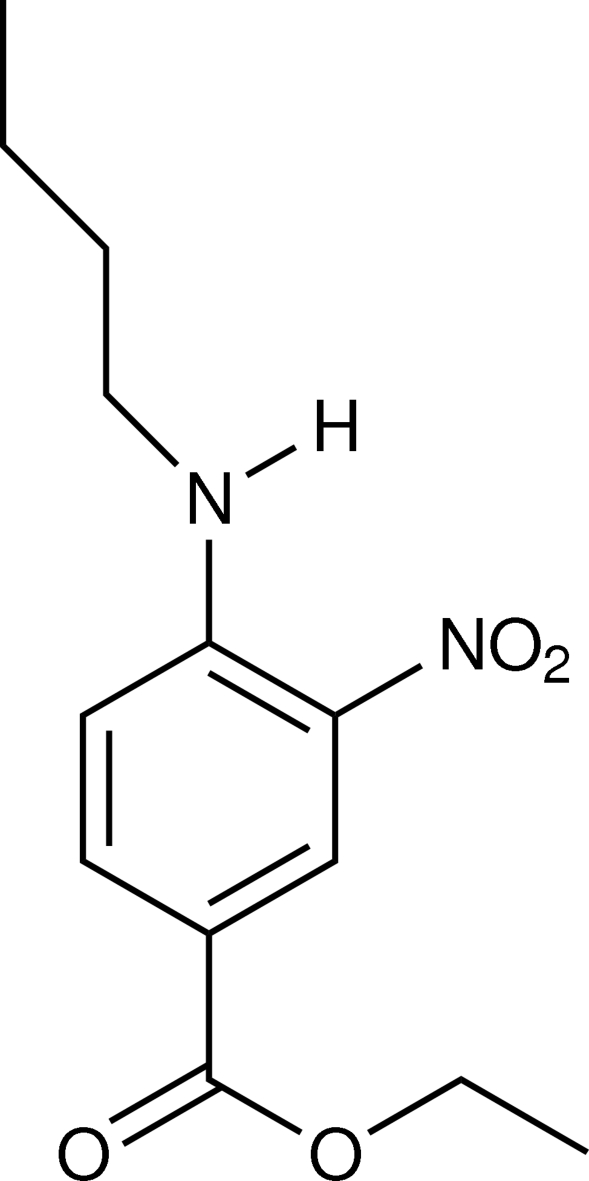


## Experimental

### 

#### Crystal data

C_13_H_18_N_2_O_4_
*M* *_r_* = 266.29
Monoclinic, 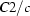

*a* = 65.292 (2) Å
*b* = 3.9555 (2) Å
*c* = 31.4417 (11) Å
β = 104.833 (3)°
*V* = 7849.6 (5) Å^3^
*Z* = 24
Mo *K*α radiation
μ = 0.10 mm^−1^
*T* = 100 K
0.40 × 0.19 × 0.03 mm

#### Data collection

Bruker SMART APEXII CCD area-detector diffractometer
Absorption correction: multi-scan (*SADABS*; Bruker, 2005 ▶) *T*_min_ = 0.915, *T*_max_ = 0.997
85498 measured reflections
8991 independent reflections
6753 reflections with *I* > 2σ(*I*)
*R*_int_ = 0.090

#### Refinement

*R*[*F* ^2^ > 2σ(*F* ^2^)] = 0.076
*wR*(*F* ^2^) = 0.177
*S* = 1.13
8991 reflections
532 parameters
H atoms treated by a mixture of independent and constrained refinement
Δρ_max_ = 0.31 e Å^−3^
Δρ_min_ = −0.25 e Å^−3^

### 

Data collection: *APEX2* (Bruker, 2005 ▶); cell refinement: *SAINT* (Bruker, 2005 ▶); data reduction: *SAINT*; program(s) used to solve structure: *SHELXTL* (Sheldrick, 2008 ▶); program(s) used to refine structure: *SHELXTL*; molecular graphics: *SHELXTL*; software used to prepare material for publication: *SHELXTL* and *PLATON* (Spek, 2009 ▶).

## Supplementary Material

Crystal structure: contains datablocks global, I. DOI: 10.1107/S1600536809029754/bq2152sup1.cif

Structure factors: contains datablocks I. DOI: 10.1107/S1600536809029754/bq2152Isup2.hkl

Additional supplementary materials:  crystallographic information; 3D view; checkCIF report

## Figures and Tables

**Table 1:** Hydrogen-bond geometry (Å, °)

*D*—H⋯*A*	*D*—H	H⋯*A*	*D*⋯*A*	*D*—H⋯*A*
N2*A*—H2*NA*⋯O4*A*^i^	0.89 (3)	2.51 (3)	3.345 (3)	156 (3)
C1*A*—H1*AA*⋯O3*A*^ii^	0.96	2.41	3.267 (3)	149
N2*B*—H2*NB*⋯O4*B*	0.83 (3)	2.02 (3)	2.637 (3)	130 (3)
N2*A*—H2*NA*⋯O4*A*	0.89 (3)	1.97 (3)	2.636 (3)	131 (3)
N2*C*—H2*NC*⋯O4*C*	0.82 (3)	2.02 (4)	2.635 (3)	132 (3)
C10*A*—H10*F*⋯O1*B*	0.97	2.58	3.542 (4)	169

Symmetry codes: (i) 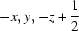; (ii) 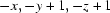.

**Fig. 1.**
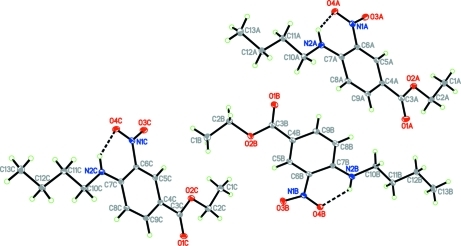
The molecular structure of (I), showing 50% probability displacement ellipsoids and the atom numbering scheme. Dashed lines indicate hydrogen bonding.

**Fig. 2.**
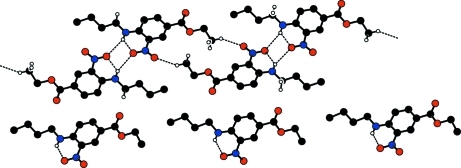
Part of the crystal packing (I). Dashed lines indicate the hydrogen bonding.

## supplementary crystallographic information

### Comment

Nitro benzoic acid derivatives are important intermediates for the synthesis of various heterocyclic compounds of pharmacological interest (Brouillette *et al.* 1999; Williams *et al.* 1995). The synthesis of novel methyl or ethyl 1*H*-benzimidazole-5-carboxylates derivatives (Ozden *et al.* 2005), non-nucleoside benzimidazole-based allosteric inhibitors of the hepatitis C virus ns5b polymerase inhibitors (Beaulieu *et al.* 2004) and solid-phase synthesis of substituted 2-aminomethyl benzimidazoles (Kilburn *et al.* 2000) were commonly accessed *via* nitrobenzoic acid derivatives. As part of an ongoing study on such compounds, in this paper, we present the crystal structure of the title compound, (I), which was synthesized as an intermediate.

The asymmetric unit of (I) consists of three crystallographically independent ethyl 4-(butylamino)-3-nitrobenzoate molecules (*A*, *B* & *C*), as shown in Fig. 1. Three intramolecular N—H···O hydrogen bonds generate S(6) ring motifs (Table 1).

In the crystal structure, in two molecules (*A* & *B*), the hydrogen atom attached to the nitrogen atom is hydrogen-bonded to the nitro group oxygen atoms *via* N—H···O hydrogen bonds to form tandem hydrogen bonds. Here both the hydrogen atoms act as bifurcated donor and the oxygen atom act as bifurcated acceptor. In addition, these neighboring molecules are linked by C—H···O hydrogen bonds along the [1 0 0] direction. Interestingly enough, the molecules *C* are not linked by any intermolecular interactions (Fig. 2).

### Experimental

The title compound was synthesized by adding *N*,*N*-diisopropyl ethylamine (DIPEA) (0.20 ml, 1.12 mmol) dropwise to a stirred solution of ethyl 4-fluoro-3-nitrobenzoate (0.21 g, 1 mmol) in dry dichloromethane (10 ml). Butylamine (0.10 ml, 1 mmol) was added slowly with stirring, and then the mixture was stirred overnight at room temperature under N_2_. After completion of the reaction, the mixture was washed with 10% Na_2_CO_3_ (10 ml). The aqueous layer was washed again with dichloromethane (3 × 10 ml). The organic fractions were pooled, dried over MgSO_4_ and the solvent was evaporated *in vacuo*. Recrystallization with hot hexane afforded the title compound as yellow needle-like crystals.

### Refinement

H atoms were positioned geometrically (C—H = 0.93–0.97 Å) and refined using a riding model with *U*_iso_(H) = 1.2*U*_eq_(C) and 1.5*U*_eq_(methyl C). A rotating–group model was used for the methyl groups. The nitrogen H atoms were located from the difference Fourier map [N–H = 0.82 (3)–0.89 (3) Å] and allowed to refine freely.

### Crystal data

**Table:**

C_13_H_18_N_2_O_4_	*F*(000) = 3408
*M**_r_* = 266.29	*D*_x_ = 1.352 Mg m^−^^3^
Monoclinic, *C*2/*c*	Mo *K*α radiation, λ = 0.71073 Å
Hall symbol: -C 2yc	Cell parameters from 8010 reflections
*a* = 65.292 (2) Å	θ = 0.00–0.00°
*b* = 3.9555 (2) Å	µ = 0.10 mm^−^^1^
*c* = 31.4417 (11) Å	*T* = 100 K
β = 104.833 (3)°	Needle, yellow
*V* = 7849.6 (5) Å^3^	0.40 × 0.19 × 0.03 mm
*Z* = 24	

### Data collection

**Table:**

Bruker SMART APEXII CCD area-detector diffractometer	8991 independent reflections
Radiation source: fine-focus sealed tube	6753 reflections with *I* > 2σ(*I*)
graphite	*R*_int_ = 0.090
φ and ω scans	θ_max_ = 27.5°, θ_min_ = 0.7°
Absorption correction: multi-scan (*SADABS*; Bruker, 2005)	*h* = −84→84
*T*_min_ = 0.915, *T*_max_ = 0.997	*k* = −5→5
85498 measured reflections	*l* = −40→40

### Refinement

**Table:**

Refinement on *F*^2^	Primary atom site location: structure-invariant direct methods
Least-squares matrix: full	Secondary atom site location: difference Fourier map
*R*[*F*^2^ > 2σ(*F*^2^)] = 0.076	Hydrogen site location: inferred from neighbouring sites
*wR*(*F*^2^) = 0.177	H atoms treated by a mixture of independent and constrained refinement
*S* = 1.13	*w* = 1/[σ^2^(*F*_o_^2^) + (0.04*P*)^2^ + 36.3175*P*] where *P* = (*F*_o_^2^ + 2*F*_c_^2^)/3
8991 reflections	(Δ/σ)_max_ < 0.001
532 parameters	Δρ_max_ = 0.31 e Å^−^^3^
0 restraints	Δρ_min_ = −0.25 e Å^−^^3^

### Special details

**Table:**

Experimental. The crystal was placed in the cold stream of an Oxford Cryosystems Cobra open-flow nitrogen cryostat (Cosier & Glazer, 1986) operating at 100.0 (1) K.
Geometry. All s.u.'s (except the s.u. in the dihedral angle between two l.s. planes) are estimated using the full covariance matrix. The cell s.u.'s are taken into account individually in the estimation of s.u.'s in distances, angles and torsion angles; correlations between s.u.'s in cell parameters are only used when they are defined by crystal symmetry. An approximate (isotropic) treatment of cell s.u.'s is used for estimating s.u.'s involving l.s. planes.
Refinement. Refinement of F^2^ against ALL reflections. The weighted R-factor wR and goodness of fit S are based on F^2^, conventional R-factors R are based on F, with F set to zero for negative F^2^. The threshold expression of F^2^ > σ(F^2^) is used only for calculating R-factors(gt) etc. and is not relevant to the choice of reflections for refinement. R-factors based on F^2^ are statistically about twice as large as those based on F, and R- factors based on ALL data will be even larger.

### Fractional atomic coordinates and isotropic or equivalent isotropic displacement parameters (Å^2^)

**Table:**

	*x*	*y*	*z*	*U*_iso_*/*U*_eq_	
O1A	0.06501 (3)	−0.0471 (6)	0.49353 (6)	0.0262 (5)	
O2A	0.03378 (3)	0.2100 (5)	0.48936 (6)	0.0201 (4)	
O3A	−0.00677 (3)	0.6630 (6)	0.35544 (6)	0.0252 (5)	
O4A	0.00171 (3)	0.6139 (6)	0.29354 (6)	0.0229 (5)	
N1A	0.00508 (4)	0.5558 (6)	0.33362 (8)	0.0188 (5)	
N2A	0.03624 (4)	0.2884 (7)	0.28855 (8)	0.0178 (5)	
C1A	0.01824 (5)	0.2944 (9)	0.54946 (9)	0.0257 (7)	
H1AA	0.0207	0.2875	0.5809	0.039*	
H1AB	0.0068	0.1440	0.5363	0.039*	
H1AC	0.0146	0.5206	0.5392	0.039*	
C2A	0.03805 (4)	0.1864 (8)	0.53679 (9)	0.0215 (6)	
H2AA	0.0498	0.3326	0.5506	0.026*	
H2AB	0.0417	−0.0440	0.5463	0.026*	
C3A	0.04914 (4)	0.0866 (8)	0.47201 (9)	0.0187 (6)	
C4A	0.04423 (4)	0.1364 (7)	0.42388 (9)	0.0170 (6)	
C5A	0.02675 (4)	0.3068 (7)	0.39999 (9)	0.0165 (6)	
H5AA	0.0169	0.3886	0.4141	0.020*	
C6A	0.02354 (4)	0.3594 (7)	0.35516 (9)	0.0169 (6)	
C7A	0.03814 (4)	0.2401 (7)	0.33173 (9)	0.0165 (6)	
C8A	0.05587 (4)	0.0581 (7)	0.35738 (9)	0.0176 (6)	
H8AA	0.0657	−0.0304	0.3436	0.021*	
C9A	0.05883 (4)	0.0099 (8)	0.40161 (9)	0.0181 (6)	
H9AA	0.0707	−0.1083	0.4173	0.022*	
C10A	0.05237 (4)	0.1805 (8)	0.26671 (9)	0.0180 (6)	
H10E	0.0547	−0.0607	0.2708	0.022*	
H10F	0.0656	0.2956	0.2798	0.022*	
C11A	0.04547 (4)	0.2598 (8)	0.21793 (9)	0.0185 (6)	
H11E	0.0416	0.4967	0.2140	0.022*	
H11F	0.0330	0.1267	0.2044	0.022*	
C12A	0.06284 (4)	0.1846 (8)	0.19501 (9)	0.0191 (6)	
H12E	0.0668	−0.0515	0.1994	0.023*	
H12F	0.0752	0.3201	0.2083	0.023*	
C13A	0.05590 (5)	0.2592 (9)	0.14579 (9)	0.0257 (7)	
H13G	0.0668	0.1890	0.1322	0.038*	
H13H	0.0535	0.4974	0.1413	0.038*	
H13I	0.0431	0.1380	0.1329	0.038*	
O1B	0.09697 (3)	0.7037 (6)	0.30897 (7)	0.0264 (5)	
O2B	0.12968 (3)	0.9272 (6)	0.32094 (6)	0.0230 (5)	
O3B	0.17262 (3)	1.0571 (6)	0.46621 (7)	0.0286 (5)	
O4B	0.16496 (3)	0.8443 (6)	0.52368 (6)	0.0248 (5)	
N1B	0.16074 (3)	0.8951 (7)	0.48336 (8)	0.0195 (5)	
N2B	0.12866 (4)	0.5300 (7)	0.51718 (8)	0.0188 (5)	
C1B	0.14493 (5)	1.1508 (9)	0.26583 (10)	0.0293 (7)	
H1BA	0.1423	1.2167	0.2355	0.044*	
H1BB	0.1557	0.9798	0.2722	0.044*	
H1BC	0.1496	1.3437	0.2843	0.044*	
C2B	0.12485 (4)	1.0126 (9)	0.27447 (9)	0.0240 (7)	
H2BA	0.1137	1.1807	0.2674	0.029*	
H2BB	0.1202	0.8133	0.2566	0.029*	
C3B	0.11389 (4)	0.7744 (8)	0.33400 (9)	0.0191 (6)	
C4B	0.11916 (4)	0.7104 (8)	0.38191 (9)	0.0183 (6)	
C5B	0.13752 (4)	0.8196 (8)	0.41074 (9)	0.0188 (6)	
H5BA	0.1476	0.9339	0.4001	0.023*	
C6B	0.14114 (4)	0.7609 (7)	0.45575 (9)	0.0169 (6)	
C7B	0.12620 (4)	0.5899 (7)	0.47410 (9)	0.0159 (6)	
C8B	0.10746 (4)	0.4822 (8)	0.44326 (9)	0.0199 (6)	
H8BA	0.0971	0.3703	0.4534	0.024*	
C9B	0.10416 (4)	0.5381 (8)	0.39906 (9)	0.0194 (6)	
H9BA	0.0917	0.4605	0.3799	0.023*	
C10B	0.11209 (4)	0.3819 (7)	0.53512 (9)	0.0174 (6)	
H10C	0.1083	0.1602	0.5223	0.021*	
H10D	0.0995	0.5234	0.5276	0.021*	
C11B	0.11981 (4)	0.3507 (8)	0.58467 (9)	0.0197 (6)	
H11C	0.1247	0.5696	0.5971	0.024*	
H11D	0.1317	0.1954	0.5919	0.024*	
C12B	0.10245 (4)	0.2244 (8)	0.60518 (9)	0.0217 (6)	
H12C	0.0973	0.0079	0.5924	0.026*	
H12D	0.0907	0.3826	0.5986	0.026*	
C13B	0.11062 (5)	0.1861 (10)	0.65495 (10)	0.0344 (8)	
H13D	0.0994	0.1052	0.6669	0.052*	
H13E	0.1155	0.4013	0.6678	0.052*	
H13F	0.1221	0.0277	0.6615	0.052*	
O1C	0.23186 (3)	1.0386 (6)	0.33479 (6)	0.0234 (5)	
O2C	0.19996 (3)	1.2945 (6)	0.31998 (6)	0.0206 (4)	
O3C	0.15980 (3)	1.4495 (6)	0.17315 (7)	0.0270 (5)	
O4C	0.16732 (3)	1.2089 (6)	0.11696 (6)	0.0241 (5)	
N1C	0.17152 (3)	1.2711 (6)	0.15709 (8)	0.0184 (5)	
N2C	0.20352 (4)	0.8875 (6)	0.12565 (8)	0.0168 (5)	
C1C	0.18335 (5)	1.5198 (9)	0.37305 (10)	0.0257 (7)	
H1CA	0.1854	1.5871	0.4032	0.039*	
H1CB	0.1723	1.3526	0.3658	0.039*	
H1CC	0.1793	1.7131	0.3543	0.039*	
C2C	0.20364 (5)	1.3733 (8)	0.36649 (9)	0.0222 (6)	
H2CA	0.2151	1.5354	0.3753	0.027*	
H2CB	0.2074	1.1703	0.3840	0.027*	
C3C	0.21578 (4)	1.1305 (7)	0.30853 (9)	0.0179 (6)	
C4C	0.21160 (4)	1.0758 (7)	0.26043 (9)	0.0162 (6)	
C5C	0.19371 (4)	1.1935 (7)	0.23052 (9)	0.0157 (5)	
H5CA	0.1836	1.3132	0.2404	0.019*	
C6C	0.19066 (4)	1.1347 (7)	0.18548 (9)	0.0157 (6)	
C7C	0.20581 (4)	0.9571 (7)	0.16860 (9)	0.0146 (5)	
C8C	0.22437 (4)	0.8497 (7)	0.20039 (9)	0.0158 (6)	
H8CA	0.2350	0.7410	0.1909	0.019*	
C9C	0.22695 (4)	0.9016 (7)	0.24435 (9)	0.0163 (6)	
H9CA	0.2391	0.8205	0.2642	0.020*	
C10C	0.22007 (4)	0.7308 (8)	0.10874 (9)	0.0177 (6)	
H10A	0.2241	0.5161	0.1234	0.021*	
H10B	0.2325	0.8756	0.1148	0.021*	
C11C	0.21220 (4)	0.6747 (8)	0.05957 (9)	0.0180 (6)	
H11A	0.2072	0.8876	0.0454	0.022*	
H11B	0.2003	0.5188	0.0539	0.022*	
C12C	0.22942 (4)	0.5331 (8)	0.03965 (9)	0.0197 (6)	
H12A	0.2347	0.3234	0.0544	0.024*	
H12B	0.2411	0.6920	0.0446	0.024*	
C13C	0.22125 (5)	0.4672 (8)	−0.00982 (9)	0.0245 (7)	
H13A	0.2325	0.3744	−0.0209	0.037*	
H13B	0.2165	0.6758	−0.0247	0.037*	
H13C	0.2097	0.3100	−0.0149	0.037*	
H2NB	0.1398 (5)	0.593 (9)	0.5348 (10)	0.020 (8)*	
H2NA	0.0249 (5)	0.402 (9)	0.2736 (11)	0.028 (9)*	
H2NC	0.1923 (5)	0.941 (10)	0.1086 (11)	0.031 (10)*	

### Atomic displacement parameters (Å^2^)

**Table:**

	*U*^11^	*U*^22^	*U*^33^	*U*^12^	*U*^13^	*U*^23^
O1A	0.0196 (10)	0.0369 (13)	0.0205 (10)	0.0087 (10)	0.0024 (8)	0.0007 (10)
O2A	0.0190 (10)	0.0275 (12)	0.0135 (9)	0.0031 (9)	0.0034 (8)	−0.0003 (8)
O3A	0.0217 (10)	0.0356 (13)	0.0196 (10)	0.0082 (10)	0.0073 (8)	0.0022 (10)
O4A	0.0197 (10)	0.0314 (13)	0.0172 (10)	0.0040 (9)	0.0043 (8)	0.0028 (9)
N1A	0.0162 (11)	0.0206 (13)	0.0207 (12)	−0.0002 (10)	0.0068 (9)	−0.0007 (10)
N2A	0.0159 (11)	0.0208 (13)	0.0171 (12)	0.0009 (10)	0.0047 (9)	0.0001 (10)
C1A	0.0283 (16)	0.0309 (18)	0.0176 (14)	0.0024 (14)	0.0054 (12)	−0.0006 (13)
C2A	0.0240 (14)	0.0269 (16)	0.0122 (13)	0.0025 (13)	0.0018 (11)	0.0018 (12)
C3A	0.0161 (13)	0.0203 (15)	0.0202 (14)	−0.0013 (11)	0.0054 (11)	−0.0033 (12)
C4A	0.0144 (13)	0.0190 (15)	0.0176 (13)	−0.0029 (11)	0.0040 (10)	−0.0005 (11)
C5A	0.0148 (12)	0.0178 (14)	0.0179 (13)	−0.0021 (11)	0.0057 (10)	−0.0006 (11)
C6A	0.0138 (12)	0.0164 (14)	0.0198 (14)	−0.0021 (11)	0.0029 (10)	−0.0006 (11)
C7A	0.0158 (13)	0.0155 (14)	0.0188 (14)	−0.0042 (11)	0.0053 (10)	−0.0026 (11)
C8A	0.0125 (12)	0.0193 (15)	0.0219 (14)	−0.0006 (11)	0.0057 (11)	−0.0035 (12)
C9A	0.0130 (12)	0.0205 (15)	0.0198 (14)	−0.0021 (11)	0.0021 (10)	0.0022 (12)
C10A	0.0175 (13)	0.0187 (15)	0.0193 (14)	0.0000 (11)	0.0073 (11)	−0.0010 (12)
C11A	0.0149 (13)	0.0219 (15)	0.0193 (14)	−0.0008 (11)	0.0055 (11)	−0.0027 (12)
C12A	0.0179 (13)	0.0187 (15)	0.0217 (14)	0.0012 (11)	0.0072 (11)	−0.0004 (12)
C13A	0.0261 (15)	0.0282 (18)	0.0242 (16)	0.0009 (13)	0.0091 (12)	−0.0009 (13)
O1B	0.0195 (10)	0.0355 (13)	0.0224 (11)	−0.0074 (10)	0.0022 (8)	−0.0008 (10)
O2B	0.0163 (10)	0.0352 (13)	0.0175 (10)	−0.0057 (9)	0.0045 (8)	0.0021 (9)
O3B	0.0199 (10)	0.0415 (14)	0.0237 (11)	−0.0123 (10)	0.0045 (9)	0.0017 (10)
O4B	0.0189 (10)	0.0381 (13)	0.0154 (10)	−0.0051 (9)	0.0008 (8)	0.0010 (9)
N1B	0.0130 (11)	0.0241 (14)	0.0210 (13)	−0.0010 (10)	0.0038 (9)	−0.0006 (10)
N2B	0.0140 (11)	0.0228 (13)	0.0191 (12)	−0.0029 (10)	0.0032 (10)	−0.0010 (10)
C1B	0.0282 (16)	0.0359 (19)	0.0233 (16)	−0.0036 (15)	0.0053 (13)	0.0032 (14)
C2B	0.0205 (14)	0.0333 (18)	0.0172 (14)	−0.0028 (13)	0.0027 (11)	0.0011 (13)
C3B	0.0162 (13)	0.0199 (15)	0.0217 (14)	−0.0008 (11)	0.0058 (11)	−0.0023 (12)
C4B	0.0143 (13)	0.0209 (15)	0.0198 (14)	−0.0005 (11)	0.0043 (11)	−0.0015 (12)
C5B	0.0141 (13)	0.0208 (15)	0.0227 (15)	−0.0007 (11)	0.0071 (11)	−0.0013 (12)
C6B	0.0128 (12)	0.0180 (14)	0.0196 (14)	0.0002 (11)	0.0036 (10)	−0.0021 (11)
C7B	0.0139 (12)	0.0166 (14)	0.0171 (13)	0.0031 (11)	0.0039 (10)	0.0004 (11)
C8B	0.0151 (13)	0.0208 (15)	0.0244 (15)	−0.0012 (11)	0.0063 (11)	−0.0029 (12)
C9B	0.0143 (13)	0.0201 (15)	0.0225 (14)	−0.0029 (11)	0.0022 (11)	−0.0049 (12)
C10B	0.0131 (12)	0.0182 (15)	0.0219 (14)	−0.0005 (11)	0.0062 (11)	0.0008 (12)
C11B	0.0174 (13)	0.0208 (15)	0.0218 (15)	−0.0011 (12)	0.0066 (11)	0.0013 (12)
C12B	0.0204 (14)	0.0261 (17)	0.0196 (14)	−0.0027 (12)	0.0067 (11)	0.0003 (12)
C13B	0.0307 (17)	0.051 (2)	0.0226 (16)	−0.0128 (17)	0.0083 (13)	0.0041 (16)
O1C	0.0148 (10)	0.0350 (13)	0.0185 (10)	0.0006 (9)	0.0007 (8)	−0.0032 (9)
O2C	0.0179 (10)	0.0288 (12)	0.0141 (9)	0.0017 (9)	0.0022 (7)	−0.0049 (9)
O3C	0.0205 (10)	0.0375 (14)	0.0228 (11)	0.0125 (10)	0.0053 (8)	0.0009 (10)
O4C	0.0192 (10)	0.0361 (13)	0.0155 (10)	0.0058 (9)	0.0016 (8)	−0.0018 (9)
N1C	0.0143 (11)	0.0222 (13)	0.0194 (12)	0.0006 (10)	0.0056 (9)	0.0006 (10)
N2C	0.0141 (11)	0.0207 (13)	0.0152 (12)	0.0015 (10)	0.0030 (9)	0.0004 (10)
C1C	0.0250 (15)	0.0306 (18)	0.0219 (15)	0.0012 (13)	0.0068 (12)	−0.0049 (13)
C2C	0.0233 (14)	0.0294 (17)	0.0121 (13)	−0.0020 (13)	0.0012 (11)	−0.0041 (12)
C3C	0.0157 (13)	0.0173 (15)	0.0204 (14)	−0.0034 (11)	0.0043 (11)	−0.0018 (11)
C4C	0.0147 (12)	0.0149 (14)	0.0188 (13)	−0.0048 (11)	0.0043 (10)	−0.0002 (11)
C5C	0.0147 (12)	0.0159 (14)	0.0175 (13)	−0.0029 (11)	0.0057 (10)	−0.0006 (11)
C6C	0.0116 (12)	0.0176 (14)	0.0165 (13)	−0.0004 (10)	0.0012 (10)	0.0037 (11)
C7C	0.0135 (12)	0.0124 (13)	0.0183 (13)	−0.0019 (10)	0.0048 (10)	0.0011 (11)
C8C	0.0119 (12)	0.0153 (14)	0.0201 (14)	0.0009 (10)	0.0038 (10)	0.0008 (11)
C9C	0.0139 (12)	0.0144 (14)	0.0189 (14)	−0.0015 (11)	0.0011 (10)	0.0009 (11)
C10C	0.0146 (12)	0.0204 (15)	0.0184 (14)	0.0015 (11)	0.0048 (10)	0.0001 (12)
C11C	0.0157 (13)	0.0200 (15)	0.0179 (14)	0.0008 (11)	0.0033 (10)	−0.0005 (12)
C12C	0.0187 (13)	0.0200 (15)	0.0214 (14)	0.0014 (12)	0.0072 (11)	−0.0001 (12)
C13C	0.0261 (15)	0.0265 (17)	0.0225 (15)	0.0039 (13)	0.0091 (12)	0.0011 (13)

### Geometric parameters (Å, °)

**Table:**

O1A—C3A	1.205 (3)	C5B—H5BA	0.9300
O2A—C3A	1.350 (3)	C6B—C7B	1.425 (4)
O2A—C2A	1.449 (3)	C7B—C8B	1.418 (4)
O3A—N1A	1.233 (3)	C8B—C9B	1.369 (4)
O4A—N1A	1.244 (3)	C8B—H8BA	0.9300
N1A—C6A	1.448 (4)	C9B—H9BA	0.9300
N2A—C7A	1.345 (3)	C10B—C11B	1.515 (4)
N2A—C10A	1.460 (3)	C10B—H10C	0.9700
N2A—H2NA	0.89 (3)	C10B—H10D	0.9700
C1A—C2A	1.510 (4)	C11B—C12B	1.524 (4)
C1A—H1AA	0.9600	C11B—H11C	0.9700
C1A—H1AB	0.9600	C11B—H11D	0.9700
C1A—H1AC	0.9600	C12B—C13B	1.526 (4)
C2A—H2AA	0.9700	C12B—H12C	0.9700
C2A—H2AB	0.9700	C12B—H12D	0.9700
C3A—C4A	1.478 (4)	C13B—H13D	0.9600
C4A—C5A	1.372 (4)	C13B—H13E	0.9600
C4A—C9A	1.411 (4)	C13B—H13F	0.9600
C5A—C6A	1.387 (4)	O1C—C3C	1.213 (3)
C5A—H5AA	0.9300	O2C—C3C	1.345 (3)
C6A—C7A	1.426 (4)	O2C—C2C	1.454 (3)
C7A—C8A	1.425 (4)	O3C—N1C	1.239 (3)
C8A—C9A	1.368 (4)	O4C—N1C	1.245 (3)
C8A—H8AA	0.9300	N1C—C6C	1.442 (3)
C9A—H9AA	0.9300	N2C—C7C	1.348 (3)
C10A—C11A	1.516 (4)	N2C—C10C	1.460 (3)
C10A—H10E	0.9700	N2C—H2NC	0.82 (3)
C10A—H10F	0.9700	C1C—C2C	1.508 (4)
C11A—C12A	1.521 (4)	C1C—H1CA	0.9600
C11A—H11E	0.9700	C1C—H1CB	0.9600
C11A—H11F	0.9700	C1C—H1CC	0.9600
C12A—C13A	1.526 (4)	C2C—H2CA	0.9700
C12A—H12E	0.9700	C2C—H2CB	0.9700
C12A—H12F	0.9700	C3C—C4C	1.482 (4)
C13A—H13G	0.9600	C4C—C5C	1.379 (4)
C13A—H13H	0.9600	C4C—C9C	1.412 (4)
C13A—H13I	0.9600	C5C—C6C	1.398 (4)
O1B—C3B	1.214 (3)	C5C—H5CA	0.9300
O2B—C3B	1.346 (3)	C6C—C7C	1.422 (4)
O2B—C2B	1.454 (3)	C7C—C8C	1.424 (4)
O3B—N1B	1.232 (3)	C8C—C9C	1.364 (4)
O4B—N1B	1.243 (3)	C8C—H8CA	0.9300
N1B—C6B	1.451 (3)	C9C—H9CA	0.9300
N2B—C7B	1.344 (3)	C10C—C11C	1.515 (4)
N2B—C10B	1.465 (3)	C10C—H10A	0.9700
N2B—H2NB	0.83 (3)	C10C—H10B	0.9700
C1B—C2B	1.508 (4)	C11C—C12C	1.526 (4)
C1B—H1BA	0.9600	C11C—H11A	0.9700
C1B—H1BB	0.9600	C11C—H11B	0.9700
C1B—H1BC	0.9600	C12C—C13C	1.532 (4)
C2B—H2BA	0.9700	C12C—H12A	0.9700
C2B—H2BB	0.9700	C12C—H12B	0.9700
C3B—C4B	1.479 (4)	C13C—H13A	0.9600
C4B—C5B	1.374 (4)	C13C—H13B	0.9600
C4B—C9B	1.409 (4)	C13C—H13C	0.9600
C5B—C6B	1.393 (4)		
			
C3A—O2A—C2A	115.2 (2)	N2B—C7B—C6B	125.0 (2)
O3A—N1A—O4A	121.8 (2)	C8B—C7B—C6B	115.2 (2)
O3A—N1A—C6A	119.3 (2)	C9B—C8B—C7B	122.0 (3)
O4A—N1A—C6A	118.9 (2)	C9B—C8B—H8BA	119.0
C7A—N2A—C10A	122.8 (2)	C7B—C8B—H8BA	119.0
C7A—N2A—H2NA	117 (2)	C8B—C9B—C4B	121.5 (3)
C10A—N2A—H2NA	120 (2)	C8B—C9B—H9BA	119.3
C2A—C1A—H1AA	109.5	C4B—C9B—H9BA	119.3
C2A—C1A—H1AB	109.5	N2B—C10B—C11B	110.1 (2)
H1AA—C1A—H1AB	109.5	N2B—C10B—H10C	109.6
C2A—C1A—H1AC	109.5	C11B—C10B—H10C	109.6
H1AA—C1A—H1AC	109.5	N2B—C10B—H10D	109.6
H1AB—C1A—H1AC	109.5	C11B—C10B—H10D	109.6
O2A—C2A—C1A	107.5 (2)	H10C—C10B—H10D	108.1
O2A—C2A—H2AA	110.2	C10B—C11B—C12B	111.9 (2)
C1A—C2A—H2AA	110.2	C10B—C11B—H11C	109.2
O2A—C2A—H2AB	110.2	C12B—C11B—H11C	109.2
C1A—C2A—H2AB	110.2	C10B—C11B—H11D	109.2
H2AA—C2A—H2AB	108.5	C12B—C11B—H11D	109.2
O1A—C3A—O2A	123.6 (3)	H11C—C11B—H11D	107.9
O1A—C3A—C4A	124.3 (3)	C11B—C12B—C13B	111.3 (2)
O2A—C3A—C4A	112.1 (2)	C11B—C12B—H12C	109.4
C5A—C4A—C9A	118.4 (3)	C13B—C12B—H12C	109.4
C5A—C4A—C3A	123.9 (2)	C11B—C12B—H12D	109.4
C9A—C4A—C3A	117.7 (2)	C13B—C12B—H12D	109.4
C4A—C5A—C6A	121.2 (3)	H12C—C12B—H12D	108.0
C4A—C5A—H5AA	119.4	C12B—C13B—H13D	109.5
C6A—C5A—H5AA	119.4	C12B—C13B—H13E	109.5
C5A—C6A—C7A	121.9 (3)	H13D—C13B—H13E	109.5
C5A—C6A—N1A	116.6 (2)	C12B—C13B—H13F	109.5
C7A—C6A—N1A	121.5 (2)	H13D—C13B—H13F	109.5
N2A—C7A—C8A	119.6 (2)	H13E—C13B—H13F	109.5
N2A—C7A—C6A	125.0 (3)	C3C—O2C—C2C	115.6 (2)
C8A—C7A—C6A	115.4 (2)	O3C—N1C—O4C	121.8 (2)
C9A—C8A—C7A	121.9 (3)	O3C—N1C—C6C	119.2 (2)
C9A—C8A—H8AA	119.0	O4C—N1C—C6C	119.0 (2)
C7A—C8A—H8AA	119.0	C7C—N2C—C10C	123.3 (2)
C8A—C9A—C4A	121.2 (3)	C7C—N2C—H2NC	117 (2)
C8A—C9A—H9AA	119.4	C10C—N2C—H2NC	120 (2)
C4A—C9A—H9AA	119.4	C2C—C1C—H1CA	109.5
N2A—C10A—C11A	110.6 (2)	C2C—C1C—H1CB	109.5
N2A—C10A—H10E	109.5	H1CA—C1C—H1CB	109.5
C11A—C10A—H10E	109.5	C2C—C1C—H1CC	109.5
N2A—C10A—H10F	109.5	H1CA—C1C—H1CC	109.5
C11A—C10A—H10F	109.5	H1CB—C1C—H1CC	109.5
H10E—C10A—H10F	108.1	O2C—C2C—C1C	107.2 (2)
C10A—C11A—C12A	112.0 (2)	O2C—C2C—H2CA	110.3
C10A—C11A—H11E	109.2	C1C—C2C—H2CA	110.3
C12A—C11A—H11E	109.2	O2C—C2C—H2CB	110.3
C10A—C11A—H11F	109.2	C1C—C2C—H2CB	110.3
C12A—C11A—H11F	109.2	H2CA—C2C—H2CB	108.5
H11E—C11A—H11F	107.9	O1C—C3C—O2C	123.5 (3)
C11A—C12A—C13A	112.3 (2)	O1C—C3C—C4C	123.5 (3)
C11A—C12A—H12E	109.2	O2C—C3C—C4C	113.0 (2)
C13A—C12A—H12E	109.2	C5C—C4C—C9C	118.4 (2)
C11A—C12A—H12F	109.2	C5C—C4C—C3C	123.3 (2)
C13A—C12A—H12F	109.2	C9C—C4C—C3C	118.3 (2)
H12E—C12A—H12F	107.9	C4C—C5C—C6C	120.7 (3)
C12A—C13A—H13G	109.5	C4C—C5C—H5CA	119.6
C12A—C13A—H13H	109.5	C6C—C5C—H5CA	119.6
H13G—C13A—H13H	109.5	C5C—C6C—C7C	121.8 (2)
C12A—C13A—H13I	109.5	C5C—C6C—N1C	116.2 (2)
H13G—C13A—H13I	109.5	C7C—C6C—N1C	122.0 (2)
H13H—C13A—H13I	109.5	N2C—C7C—C6C	124.4 (2)
C3B—O2B—C2B	115.1 (2)	N2C—C7C—C8C	119.9 (2)
O3B—N1B—O4B	122.0 (2)	C6C—C7C—C8C	115.7 (2)
O3B—N1B—C6B	119.1 (2)	C9C—C8C—C7C	121.9 (2)
O4B—N1B—C6B	118.9 (2)	C9C—C8C—H8CA	119.1
C7B—N2B—C10B	123.3 (2)	C7C—C8C—H8CA	119.1
C7B—N2B—H2NB	118 (2)	C8C—C9C—C4C	121.4 (2)
C10B—N2B—H2NB	118 (2)	C8C—C9C—H9CA	119.3
C2B—C1B—H1BA	109.5	C4C—C9C—H9CA	119.3
C2B—C1B—H1BB	109.5	N2C—C10C—C11C	110.2 (2)
H1BA—C1B—H1BB	109.5	N2C—C10C—H10A	109.6
C2B—C1B—H1BC	109.5	C11C—C10C—H10A	109.6
H1BA—C1B—H1BC	109.5	N2C—C10C—H10B	109.6
H1BB—C1B—H1BC	109.5	C11C—C10C—H10B	109.6
O2B—C2B—C1B	106.9 (2)	H10A—C10C—H10B	108.1
O2B—C2B—H2BA	110.3	C10C—C11C—C12C	112.3 (2)
C1B—C2B—H2BA	110.3	C10C—C11C—H11A	109.1
O2B—C2B—H2BB	110.3	C12C—C11C—H11A	109.1
C1B—C2B—H2BB	110.3	C10C—C11C—H11B	109.1
H2BA—C2B—H2BB	108.6	C12C—C11C—H11B	109.1
O1B—C3B—O2B	123.1 (3)	H11A—C11C—H11B	107.9
O1B—C3B—C4B	123.8 (3)	C11C—C12C—C13C	112.2 (2)
O2B—C3B—C4B	113.0 (2)	C11C—C12C—H12A	109.2
C5B—C4B—C9B	118.3 (3)	C13C—C12C—H12A	109.2
C5B—C4B—C3B	123.7 (3)	C11C—C12C—H12B	109.2
C9B—C4B—C3B	118.0 (2)	C13C—C12C—H12B	109.2
C4B—C5B—C6B	120.7 (3)	H12A—C12C—H12B	107.9
C4B—C5B—H5BA	119.7	C12C—C13C—H13A	109.5
C6B—C5B—H5BA	119.7	C12C—C13C—H13B	109.5
C5B—C6B—C7B	122.3 (2)	H13A—C13C—H13B	109.5
C5B—C6B—N1B	116.2 (2)	C12C—C13C—H13C	109.5
C7B—C6B—N1B	121.5 (2)	H13A—C13C—H13C	109.5
N2B—C7B—C8B	119.7 (3)	H13B—C13C—H13C	109.5
			
C3A—O2A—C2A—C1A	−173.0 (3)	C10B—N2B—C7B—C8B	−5.3 (4)
C2A—O2A—C3A—O1A	2.9 (4)	C10B—N2B—C7B—C6B	174.0 (3)
C2A—O2A—C3A—C4A	−176.8 (2)	C5B—C6B—C7B—N2B	−179.0 (3)
O1A—C3A—C4A—C5A	−175.9 (3)	N1B—C6B—C7B—N2B	−1.3 (4)
O2A—C3A—C4A—C5A	3.8 (4)	C5B—C6B—C7B—C8B	0.3 (4)
O1A—C3A—C4A—C9A	2.0 (5)	N1B—C6B—C7B—C8B	178.0 (3)
O2A—C3A—C4A—C9A	−178.3 (3)	N2B—C7B—C8B—C9B	179.8 (3)
C9A—C4A—C5A—C6A	−1.0 (4)	C6B—C7B—C8B—C9B	0.4 (4)
C3A—C4A—C5A—C6A	176.9 (3)	C7B—C8B—C9B—C4B	−0.9 (5)
C4A—C5A—C6A—C7A	0.0 (4)	C5B—C4B—C9B—C8B	0.7 (4)
C4A—C5A—C6A—N1A	−177.2 (3)	C3B—C4B—C9B—C8B	−177.1 (3)
O3A—N1A—C6A—C5A	−0.5 (4)	C7B—N2B—C10B—C11B	−179.5 (3)
O4A—N1A—C6A—C5A	178.9 (3)	N2B—C10B—C11B—C12B	175.3 (2)
O3A—N1A—C6A—C7A	−177.7 (3)	C10B—C11B—C12B—C13B	178.6 (3)
O4A—N1A—C6A—C7A	1.7 (4)	C3C—O2C—C2C—C1C	−173.9 (3)
C10A—N2A—C7A—C8A	−4.0 (4)	C2C—O2C—C3C—O1C	3.0 (4)
C10A—N2A—C7A—C6A	175.8 (3)	C2C—O2C—C3C—C4C	−176.6 (2)
C5A—C6A—C7A—N2A	−178.5 (3)	O1C—C3C—C4C—C5C	−177.1 (3)
N1A—C6A—C7A—N2A	−1.4 (4)	O2C—C3C—C4C—C5C	2.5 (4)
C5A—C6A—C7A—C8A	1.3 (4)	O1C—C3C—C4C—C9C	1.3 (4)
N1A—C6A—C7A—C8A	178.3 (2)	O2C—C3C—C4C—C9C	−179.1 (2)
N2A—C7A—C8A—C9A	178.2 (3)	C9C—C4C—C5C—C6C	1.4 (4)
C6A—C7A—C8A—C9A	−1.6 (4)	C3C—C4C—C5C—C6C	179.8 (3)
C7A—C8A—C9A—C4A	0.6 (4)	C4C—C5C—C6C—C7C	−0.9 (4)
C5A—C4A—C9A—C8A	0.7 (4)	C4C—C5C—C6C—N1C	−179.5 (3)
C3A—C4A—C9A—C8A	−177.3 (3)	O3C—N1C—C6C—C5C	5.1 (4)
C7A—N2A—C10A—C11A	178.3 (3)	O4C—N1C—C6C—C5C	−175.6 (2)
N2A—C10A—C11A—C12A	174.0 (2)	O3C—N1C—C6C—C7C	−173.6 (3)
C10A—C11A—C12A—C13A	179.2 (3)	O4C—N1C—C6C—C7C	5.8 (4)
C3B—O2B—C2B—C1B	−175.0 (3)	C10C—N2C—C7C—C6C	174.5 (3)
C2B—O2B—C3B—O1B	1.8 (4)	C10C—N2C—C7C—C8C	−5.7 (4)
C2B—O2B—C3B—C4B	−176.7 (2)	C5C—C6C—C7C—N2C	178.7 (3)
O1B—C3B—C4B—C5B	−173.5 (3)	N1C—C6C—C7C—N2C	−2.8 (4)
O2B—C3B—C4B—C5B	5.0 (4)	C5C—C6C—C7C—C8C	−1.2 (4)
O1B—C3B—C4B—C9B	4.2 (5)	N1C—C6C—C7C—C8C	177.4 (2)
O2B—C3B—C4B—C9B	−177.3 (3)	N2C—C7C—C8C—C9C	−177.1 (3)
C9B—C4B—C5B—C6B	0.0 (4)	C6C—C7C—C8C—C9C	2.7 (4)
C3B—C4B—C5B—C6B	177.7 (3)	C7C—C8C—C9C—C4C	−2.3 (4)
C4B—C5B—C6B—C7B	−0.5 (4)	C5C—C4C—C9C—C8C	0.1 (4)
C4B—C5B—C6B—N1B	−178.3 (3)	C3C—C4C—C9C—C8C	−178.4 (3)
O3B—N1B—C6B—C5B	1.8 (4)	C7C—N2C—C10C—C11C	177.8 (3)
O4B—N1B—C6B—C5B	−178.6 (3)	N2C—C10C—C11C—C12C	176.1 (2)
O3B—N1B—C6B—C7B	−176.1 (3)	C10C—C11C—C12C—C13C	178.4 (2)
O4B—N1B—C6B—C7B	3.6 (4)		

### Hydrogen-bond geometry (Å, °)

**Table:**

*D*—H···*A*	*D*—H	H···*A*	*D*···*A*	*D*—H···*A*
N2A—H2NA···O4A^i^	0.89 (3)	2.51 (3)	3.345 (3)	156 (3)
C1A—H1AA···O3A^ii^	0.96	2.41	3.267 (3)	149
N2B—H2NB···O4B	0.83 (3)	2.02 (3)	2.637 (3)	130 (3)
N2A—H2NA···O4A	0.89 (3)	1.97 (3)	2.636 (3)	131 (3)
N2C—H2NC···O4C	0.82 (3)	2.02 (4)	2.635 (3)	132 (3)
C10A—H10F···O1B	0.97	2.58	3.542 (4)	169

Symmetry codes: (i) −*x*, *y*, −*z*+1/2; (ii) −*x*, −*y*+1, −*z*+1.
